# Altered expression of the mismatch repair genes in DF-1 cells infected with the avian leukosis virus subgroup A

**DOI:** 10.1186/s40064-016-3433-5

**Published:** 2016-10-07

**Authors:** Da-wei Yao, Li Zhan, Yu-fang Hong, Jian-xin Liu, Jia-rong Xu, De-ji Yang

**Affiliations:** College of Veterinary Medicine, Nanjing Agricultural University, Nanjing, 210095 Jiangsu China

**Keywords:** ALV, DF-1 cells, Microsatellite instability, Mismatch repair

## Abstract

The absence or deficiency of DNA mismatch repair (MMR) activity results in microsatellite instability (MSI) in cancer. The avian leukosis virus (ALV) causes neoplastic disease in chickens. In this study, the status of MMR, MSI, the cell cycle and apoptosis were detected in DF-1 cells after avian leukosis virus subgroup A infection. Flow cytometry analysis results indicated that there was no significant difference in cell apoptosis between the control and infected groups. The percentage of cells in S and G2 phases were increased in the infected group. MSI and mutation of MSH2 and MLH1 gene exons were absent in DF-1 cells after infection. Levels of MSH2 and MLH1 mRNA were dramatically increased in DF-1 cells after infection. These results demonstrated that ALV RAV-1 infection may promote the expression of MSH2 and MLH1 genes rather than resulting in gene mutations. Mismatch repair functions were normal and may be have relationships with the arrest of S phase and G2 phase.

## Background

DNA mismatch repair (MMR) is a highly conserved pathway from yeast to humans (Klucking et al. 2005). It contributes to DNA replication fidelity by recognizing mis incorporation errors and facilitating their excision. Several MMR proteins have been identified in eukaryotic cells include the MutS homologs (MutSα and MutSβ) and the MutL homologs (MutLα and MutLβ) (Hsieh and Yamane 2008). MutSα, MSH2–MSH6, recognizes single base mismatches and small loops in the DNA. MutSβ, MSH2-MSH3, recognizes lager loops. MLH1 and PMS2 bind to form MutL protein, known as MutLα, in mammalian cells. The formation of a ternary complex consisting of MutSα, MutLα and a mismatched DNA signals the start of the excision step of MMR. The following steps include strand discrimination, excision, resynthesis, and ligation by exonuclease I (EXO1), proliferating cell nuclear antigen (PCNA), replication protein A (RPA), single strand DNA-binding protein (SSB), high mobility group protein B1(HMGB1), DNA pol δ, DNA ligase I and so on.

The absence or deficiency of DNA MMR activity can developed instability in simple sequence repeats, that known as microsatellite instability (MSI). MSI can be detected by isolation of the DNA from a tumor specimen that has defective DNA MMR activity. The microsatellite sequence was amplified by polymerase chain reaction (PCR), and compared with normal DNA of that individual. The DNA of the cancerous cell contains deletions or addition in the microsatellite sequence, which can serve as a surrogate for the absence of DNA MMR activity. Some microsatellite sequences occur in coding regions and mutations in target genes actually mediate neoplastic behavior, This phenotype was first found in Lynch syndrome tumors (Boland et al. 1998). Germline mutations in one of the four major HNPCC-associated MMR genes; i.e., MLH1, MSH2, MSH6, and PMS2, are detected in up to 70–80 % of such families (Peltomaki 2003). MMR deficiency is a common feature of many types of tumors i.e.. Loss of expression from one of the MMR proteins (MSH2, MSH6, MSH3, MLH1, and PMS2) which is associated with an increased predisposition to colorectal, gastric, endometrial, ovarian, and other cancers (Guillotin and Martin 2014; McConechy et al. 2015; Shilpa et al. 2014; van Grieken et al. 2013). MSI is also observed in a subset of tumors induced with viral infections. EBV DNase caused chromosomal aberrations and increased the MSI and frequency of genetic mutations in human epithelial cells (Wu et al. 2010). The hepatitis C virus core protein promoted microsatellite instability in PH5CH8 cells. MSI was a real event occurring in primary chicken embryo fibroblasts and in chickens with lymphoma infected with MDV (Yao et al. 2013; Zhou et al. 2012).

The avian leukosis virus (ALVs), which belong to the genus alpha retrovirus of the *Retroviridae* family, cause neoplastic diseases and other reproduction problems in the poultry industry worldwide (Gao et al. 2012). According to the host range, the antigen structure of glycoproteins on the virus envelope and the interference between different strains in cell culture, avian leukosis viruses are divided into 10 subgroups (A-J), with six subgroups (A, B, C, D, E and J) existing in chickens (Dai et al. 2015; Reinisova et al. 2008; Yang et al. 2011). The subgroups A, B and J are mainly exogenous avian leukosis viruses that cause avian tumors, while subgroup E is a non-pathogenic or low pathogenic endogenous avian leukosis virus (Dai et al. 2015).

In this paper, we address the question of whether MMR deficiency and MSI are present in avian tumors induced with avian leukosis virus subgroup A. We describe the status of MSI, the MMR system (MSH2 and MLH1), the cell cycle and apoptosis in DF-1 cells infected with avian leukosis virus subgroup A. Our data revealed that infection with ALV RAV-1 could cause confusion in the cell cycle and increase the expression of MSH2 and MLH1 mRNA. There was no occurrence of MSI on the selected 16 microsatellite sites and no mutations of exons in the MSH2 and MLH1 genes.

## Methods

### Cells

DF-1 cells were grown in 25 cm^2^ flasks with Dulbecco’s modified Eagle medium (DMEM) (Thermo Fisher Scientific Inc.) supplemented with 10 % fetal bovine serum (FBS) and 1 % antibiotics (penicillin, streptomycin) and were maintained at 37 °C in an incubator under an atmosphere of 5 % CO_2_.

### Virus

Avian leukosis virus subgroup A, ALV RAV-1(CAVCC AV228), was purchased from the China Veterinary Culture Collection Center. After expanding the culture of the DF-1 cell line in our laboratory, each 0.1 mL cell supernatant contained 10^4.75^ TCID_50_.

### Infection of DF-1 with ALV RAV-1

The DF-1 cells were seeded (approximately 1 × 10^5^ cells/well) in 6-well culture plates. The cells of the infected group were infected with 100 μL ALV RAV-1 (n = 3). After 2 h incubation, the cells were washed and maintained in media with 1 % FBS for 7 days. The cells of the control group were supplemented with 100 μL DMEM at the same time (n = 3).

### Specific p27 antigen test

Samples of 150 µL of cell supernatants were collected at 7 days post infection (d.p.i.), and ALV-p27 antigen was detected with an Avian Leukosis Virus Antigen Test Kit (IDEXX USA Inc.) according to the manufacturer’s instructions.

### Cell cycle and apoptosis analysis by flow cytometry (FCM)

For cell cycle analysis, cells at 7 days post infection were fixed with ice cold 75 % ethanol and were treated with RNase A (50 μg/mL) and 0.05 % Triton X for 20 min at room temperature. Subsequently, PI (50 μg/mL) was added to the RNase treated cell suspension and incubated for 15 min in the dark before analysis with FCM (BD FACS Calibur, BD, USA). Data were analyzed using Cell Quest Pro Software. Cells were washed twice with ice-cold PBS. The levels of apoptosis were determined by flow cytometric method after Annexin V-FITC/propidium iodide (PI) double staining using an Annexin V-FITC Apoptosis Detection Kit (KeyGEN BioTECH, China) according to the manufacturer’s instructions.

### Detection of microsatellite instability

Cells were harvested at 7 days post infection (d.p.i.). DNA extraction and PCR amplification were performed according to previous reports (Zhou et al. 2012). The 16 microsatellite markers used in this research were ABR0008, ABR0547, ABR0161, ABR0587, ABR0520, ABR0197, ABR0236, ABR0563, ABR0189, ABR0579, MCW0311, MCW0027, MCW0220, ADL0217, LEI0223, and LEI0096. The primers information were reported previously (Yao et al. 2013).

### Detection of exon mutations of the mismatch repair gene

The 16 pairs of primers used for amplification of the 16 exons of the MSH2 gene were designed using Primer Premier 5.0 software and related sequence information available in the GenBank database (NC_006090). Nineteen primer pairs were designed to amplify the 19 exons of the MLH1 gene (NC_006089). The primer information is shown in Tables 1 and 2. Cell DNA was extracted and used for PCR amplification 7 days after infection. The 5 μL PCR products and 15 μL formamide denaturing buffer were mixed and denatured at 95 °C for 5 min and then bathed immediately in ice for 10 min. Samples (4 μL) were loaded on non-denaturing polyacrylamide gels to analyze gene exon mutations. After electrophoresis, the gels were stained with AgNO_3_ according to the method of Sanguinetti et al.

**Table 1:** Primers used to amplify exon of MSH2 gene

Primer	Sequence (5′–3′)	Size of fragments amplified (bp)
MSH2 exon1 F	Ggtgctgtgctgtgctgt	343
MSH2 exon1 R	Aggccatcgtgagtcaatc	
MSH2 exon2 F	Ttacaagaataaagcagggag	171
MSH2 exon2 R	Taccaccagaggcagtca	
MSH2 exon3 F	Gccaacaatgatatgtcaatg	342
MSH2 exon3 R	Ctgaacgacctgtttgcc	
MSH2 exon4 F	Gaggaaaggagaacaaatg	153
MSH2 exon4 R	Cagaacagggtcaggaag	
MSH2 exon5 F	Tgaactgactacttttgatc	182
MSH2 exon5 R	Aagcagaagctgacatt	
MSH2 exon6 F	Atcaaacagccacttatg	137
MSH2 exon6 R	Gatttcttggttgtagtatt	
MSH2 exon7 F	Ccagatcttaaccggcta	222
MSH2 exon7 R	Gaagtacacaggaaaaaacg	
MSH2 exon8 F	Gatgatagaaacaaccct	119
MSH2 exon8 R	Attagtttagtgagtgcg	
MSH2 exon9 F	Gcagaccttattgaagagt	148
MSH2 exon9 R	Gaacaacagaaacatccc	
MSH2 exon10 F	Aggttctcaggaacaacat	178
MSH2 exon10 R	Ttcagccactcaagatgt	
MSH2 exon11 F	Tcgcttcaggtgagtatg	107
MSH2 exon11 R	Ggaaggttaaggtcctactc	
MSH2 exon12 F	Tgccattgtcagctttgc	293
MSH2 exon12 R	Cctcccctcccttttgtt	
MSH2 exon13 F	Ataaccatcgtggattgta	202
MSH2 exon13 R	Aagatgctaaaagaaaatgg	
MSH2 exon14 F	Gctatttcagaatacattgct	263
MSH2 exon14 R	Agcttggagtactacacatga	
MSH2 exon15 F	Aagcactggagctggagg	178
MSH2 exon15 R	Tctggggaaatctgagtcg	
MSH2 exon16 F	Agaagacatcaaaaccaag	449
MSH2 exon16 R	Gctgtatgctatctgaagg

**Table 2:** Primers used to amplify exon of MLH1 gene

Primer	Sequence (5′–3′)	Size of fragments amplified (bp)
MLH1 exon1 F	Tctagctccaaggccact	395
MLH1 exon1 R	Actaagccgacacccatt	
MLH1 exon2 F	Ccaaccatccacctactg	326
MLH1 exon2 R	Ttactcaccctgataccaca	
MLH1 exon3 F	Cagcctaagccttctatgat	172
MLH1 exon3 R	Acctcacccctaaaacca	
MLH1 exon4 F	Ctctactgacctgctgttttc	280
MLH1 exon4 R	Aatccgttcactaccaccta	
MLH1 exon5 F	Agcagaaggtgggga	274
MLH1 exon5 R	Gggaatagtaggagaac	
MLH1 exon6 F	Cccagtgatgtggatgat	396
MLH1 exon6 R	Tggcaaaaggaattagagc	
MLH1 exon7, 8 F	Tgtagcccttcattccttt	389
MLH1 exon7, 8 R	Ttagcacagcttgcgttc	
MLH1 exon9 F	Acttgtgagggctgtttg	159
MLH1 exon9 R	Cagagtagttggcattcgt	
MLH1 exon10 F	Tgaaggattagttaggcacc	334
MLH1 exon10 R	Caaccagccttgtcacct	
MLH1 exon11 F	Tcactaatacgaatgaaccg	500
MLH1 exon11 R	Gctttgtagagggctgtg	
MLH1 exon12 F	Agtaagatgctgtgagaaacga	477
MLH1 exon12 R	Ggatgagacttaccgaggaa	
MLH1 exon13 F	Atggaagaagacaacagaaagg	141
MLH1 exon13 R	Acccgagaagcaaaacac	
MLH1 exon14 F	Atttggacagacttagcacg	212
MLH1 exon14 R	Tcatcttggaaacaacagc	
MLH1 exon15 F	Tgttgtttccaagatgacc	160
MLH1 exon15 R	Agtctcagaacgccaaag	
MLH1 exon16 F	Tgcttgctttagaggacc	200
MLH1 exon16 R	Attgctattaccagatgcc	
MLH1 exon17 F	Accaccatccttttcc	381
MLH1 exon17 R	Ctatttcagcccctcctt	
MLH1 exon18 F	Caggtaaactgggatgaaga	220
MLH1 exon18 R	Ctgaaagcagatttgggatt	
MLH1 exon19 F	Atggaaatggacagtggag	122
MLH1 exon19 R	Ctttatacaagtcaggcaggtt	

### Mismatch repair-related gene expression

Total cellular RNA was isolated from DF-1 cells 7 days after infection using RNAiso Plus (Takara, Japan), following the manufacturer’s instructions. First-strand cDNA was synthesized using PrimeScript™ RT Master Mix (Takara, Japan). The β-actin gene was used as an internal control for real-time PCR. The primers of the two main mismatch repair genes (MSH2 and MLH1) and the β-actin gene used for real-time PCR are listed in Table 3. Real-time PCR was performed with an Applied Biosystems 7500 Real-time PCR System (Applied Biosystems, USA) with a SYBR Premix Ex Taq Kit (Takara, Japan). The program were performed at 95 °C for 30 s, forty cycles of amplification consisting of denaturation at 95 °C for 5 s, annealing at 60 °C for 34 s, followed by melting curve analysis. The data were analyzed with the 2^−∆∆Ct^ method. Statistical analysis was performed by one-way analysis of variance (ANOVA) using Predictive Analytics Software 18.0. Duncan’s multiple-range test was used, with differences considered to be significant at *P* < 0.05.

**Table 3:** The primers used for real-time PCR

Primer	Sequence (5′–3′)	Size of fragments amplified (bp)
β-acting mRNA F	Gagaattgtgcgtgacatca	152
β-acting mRNA R	Cctgaacctctcattgcca	
MSH2 mRNA F	Ttacaggactgttaccgaatg	216
MSH2 mRNA R	Agccttaaccaggaactca	
MLH1 mRNA F	Acttgtgagggctgtttg	159
MLH1 mRNA R	Cagagtagttggcattcgt	

## Results

### Cell supernatant p27 antigen S/P value

Viral infection was confirmed by measuring the ALV-p27 antigen levels with an ELISA. DF-1 cells were infected with ALV RAV-1 and maintained in culture for 7 days. The cell supernatant was collected at 7 d.p.i. The S/P values of the infected group were between 0.551 and 0.920 (positive critical value was 0.2), and the S/P values of the control group were between 0.065 and 0.153. If the S/P value was more than the critical value 0.2, the p27 antigen was positive.

### Cell cycle and apoptosis results

To explore the effect of infection with ALV RAV-1 on the cell cycle and apoptosis of DF-1 cells, FCM was used. As shown in Table 4, infection with ALV RAV-1 decreased the percentage of cells in G1 phase compared with the controls (*P* < 0.05), and increased the percentage of cells in S phase and G2 phase. This result indicates that infection with ALV RAV-1 can cause confusion in the cell cycle and block DNA synthesis. As shown in Table 5, apoptosis was detected in approximately 10.00 % of the control group and 10.62 % of the infected group; there was no significant difference between the control and infected groups (*P* > 0.05). This suggests that ALV RAV-1 was not able to induce apoptosis in DF-1 cells.

**Table 4:** The results of cell cycle

Group	G1 (%)	S (%)	G2 (%)
Control (n = 3)	82.17 ± 1.37^a^	4.06 ± 0.46^b^	13.77 ± 0.93^b^
Infected (n = 3)	78.48 ± 1.49^b^	5.03 ± 0.43^a^	16.48 ± 1.36^a^

Means within a column with different superscripts are different at *P* < 0.05

**Table 5:** The results of the apoptosis rate

Group	Apoptosis (%)
Control (n = 3)	10.00 ± 0.51^a^
Infected (n = 3)	10.62 ± 0.93^a^

Means within a column with same superscripts are not significantly different

### Microsatellite instability test results

**Fig. 1:**
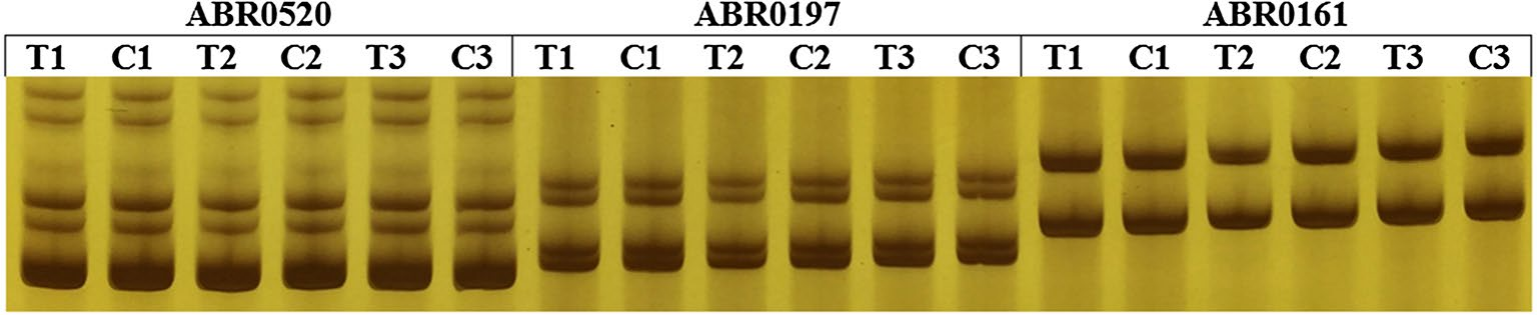
DNA extracted from normal DF-1 cells and DF-1 cells infected with ALV-A. Microsatellite markers were amplified by PCR, and the PCR products were analyzed with 12 % denaturing polyacrylamide gels (stained with AgNO_3_). The microsatellite loci ABR0520 ABR0197, ABR0161 were shown in this figure. In comparison with the control group, there were no alterations in the electrophoretic migration of major bands in the infected group. (*T* infected group, *C* control group, n = 3) (partial results)

PCR products amplified from the 16 microsatellite markers were analyzed with a denaturing polyacrylamide gel. As shown in Fig. 1, there were no differences in the electrophoretic migration of major bands between the infection group and control group. This suggests that there no changes in the genomic DNA in the microsatellite marker sites.

### Detection of mutation of MSH2 and MLH1 gene exons

**Fig. 2:**
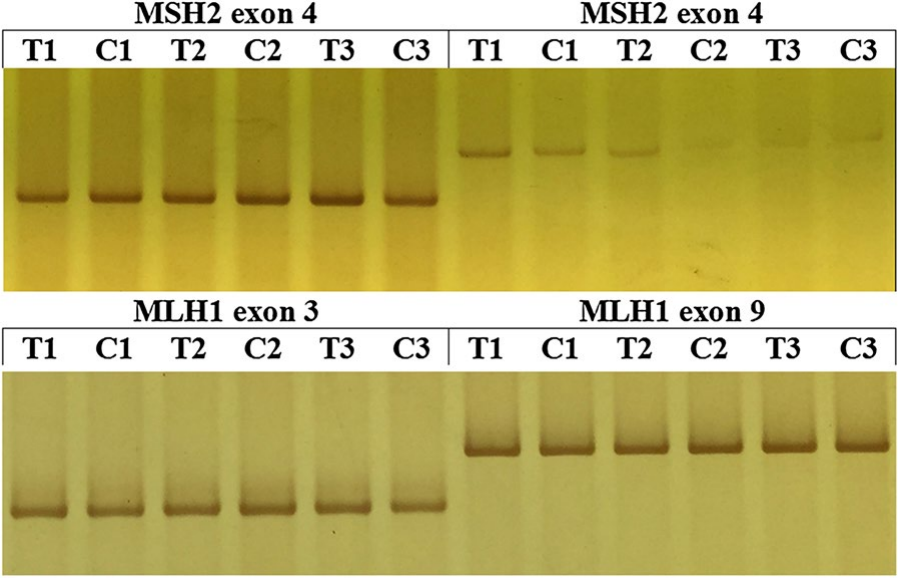
PCR products amplified from 16 MSH2 gene exons and 19 MLH1 gene exons were analyzed by non-denaturing polyacrylamide gels (partial results). In comparison with the control group, there were no alterations in the electrophoretic migration of major bands in the infected group. (*T* infected group, *C* control group, n = 3) (partial results)

The 16 exons of the MSH2 gene and 19 exons of the MLH1 gene were amplified by PCR, and then loaded onto non-denaturing polyacrylamide gels for electrophoresis. As shown in Fig. 2, there were no differences in bands between the infection group and the control group. These results suggest that no mutation was present in the MSH2 or MLH1 gene exons in DF-1 cells infected with ALV RAV-1.

### Mismatch repair-related gene expression

**Fig. 3:**
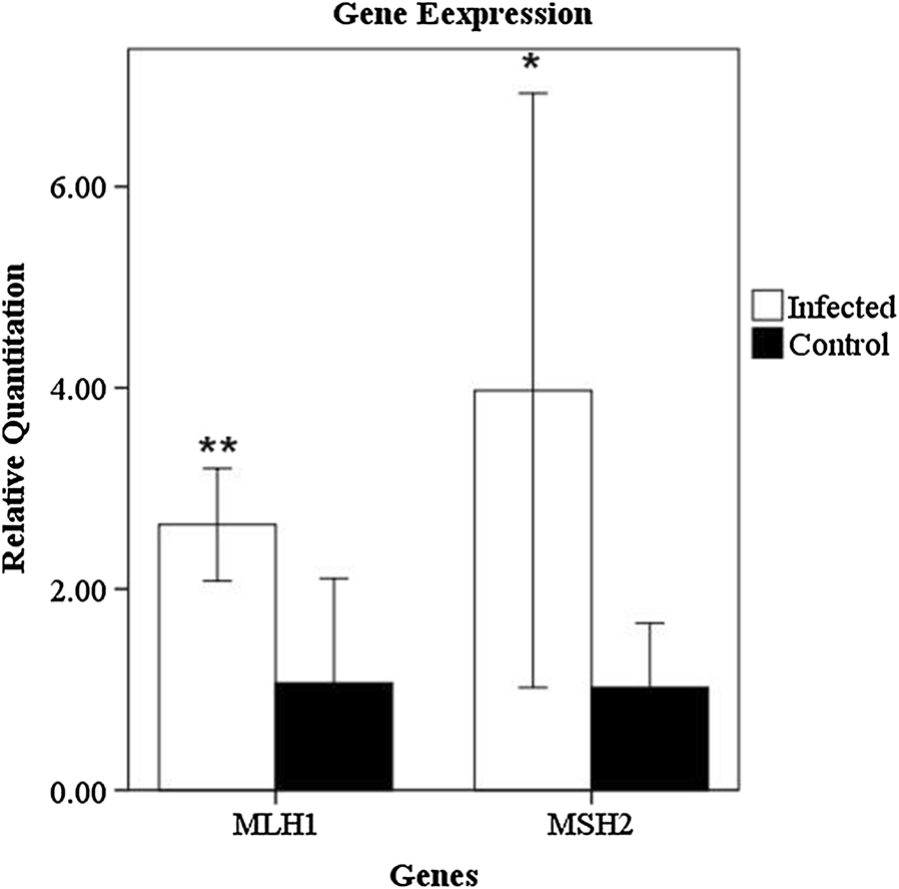
The relative expression levels of MSH2 and MLH1 mRNA in DF-1 cells infected with ALV RAV-1 were analyzed with the 2^−∆∆Ct^ method. MSH2 and MLH1 mRNA expressions of DF-1 cells infected with ALV RAV-1 were significantly increased compared to the control group (*P < 0.05, **P < 0.01). (n = 3)

As shown in Fig. 3, the MSH2 mRNA expression level of the infected group was increased fourfold compared with the control group. The MLH1 mRNA expression level was 2.5 times higher than the control group. MSH2 and MLH1 mRNA expressions of DF-1 cells infected with ALV RAV-1 were significantly increased compared with the control group *(P* < 0.05).

## Discussion

DF-1 cells are a chicken fibroblast cell line. DF-1 cells were derived from ELL chicken embryos and appeared to have a fibrous form, containing no avian leukosis virus endogenous genes (Himly et al. 1998). Endogenous ALVs could not proliferate in DF-1 cells. Therefore, we selected this cell line to reduce the effects of endogenous factors and further decrease the false positive rate in this test.

ALV can replicate and proliferate in DF-1 cells without cytopathic effects. P27 antigen is a specific antigen of the avian leukosis virus group (Yun et al. 2013). It can be produced by both exogenous viruses and endogenous viruses in infected cells, and there are no differences in the p27 antigens of different subgroups, except for the expression levels. A commercialized p27 antigen ELISA kit was used in this study to confirm infection with ALV RAV-1. ALV RAV-1 was used to infect DF-1 cells and the culture was maintained for 7 days. The S/P value of the infected group was higher than the critical value 0.2, which suggests that ALV RAV-1 successfully infected the DF-1 cells.

Most previous studies reported that there were no obvious cytopathic effects in CEFs or DF-1 cells after ALV-A infection. DF-1 cells overexpressed TVA, a low-density lipoprotein receptor-related protein, become susceptible to ALV-A induced CPE. ALV-A cytopathic effects is associated with higher levels of viral superinfection and DNA accumulation(Kolodner and Marsischky 1999). In this study, flow cytometry was utilized to evaluate DF-1 cells apoptosis. The results revealed that ALV RAV-1 did not induce cell apoptosis. Gai et al. reported ALV-A and Radish anthocyanin had a significant impact on promoting the biological activity of DF-1 cells using an MTT assay (Lili 2012). These results were contrary to previous reports. Wang et al. reported that ALV-A induced cell apoptosis in vitro using an MTT assay. The death of cells induced by ALV-A can be inhibited by adding anthocyanins from purple corn (Wang et al. 2014). In addition, it has also been reported that different results were observed in immune cells. It is likely that ALV-J infection induced apoptosis in dendritic cells (Liu et al. 2016).

Although ALV RAV-1 infection could not induce apoptosis in DF-1 cells, the flow cytometry cell cycle analysis indicated that ALV RAV-1 infection can inhibit cell cycle progression. The percentages of cells in S phase and G2 phase were increased after ALV RAV-1 infection. This suggests that cell cycle arrest occurred at S phase and G2 phase. In a previous study, a time- and dose-dependent G2/M arrest after Na_2_SeO_3_ and MSeA exposure was reported in HCT116 + hMLH1 cells, but not in HCT116 cells (Qi et al. 2010). This suggested that G2 arrest was dependent on hMLH1 and was regulated by ATM and ROS. In this study, the real-time PCR results indicate that expression of the DNA mismatch repair genes mRNA (MSH2 and MLH1) were increased after ALV RAV-1 infection. Up-regulation of MSH2 and MLH1 mRNA may have certain relationships with cycle of arrest S phase and G2 phase, and with the DNA repair of damage in DF-1 cells after ALV RAV-1 infection.

Defective MMR results in abnormalities in the processes of DNA repair. The differential instability at microsatellites emphasizes the heterogeneity of phenotypes associated with MMR. Exon mutations of MSH2 and MLH1 genes were not detected by non-denaturing polyacrylamide gel electrophoresis. The results indicate no mutation was found in MSH2 and MLH1 gene exons in DF-1 cells infected with ALV RAV-1. Meanwhile, there was no MSI of the 16 microsatellite loci in the genomic DNA of DF-1 cells infected with ALV RAV-1. These results suggest that the function of MMR in DF-1 cells infected with ALV RAV-1 was normal.

 In conclusion, our results indicate that ALV RAV-1 infection may promote the expression of MSH2 and MLH1 mRNA rather than causing gene mutations. The DF-1 cells were arrested in S phase and G2 phase after the ALV RAV-1 infection.
